# Fresh-frozen navicular allograft for a navicular osteochondral lesion: A case report

**DOI:** 10.1097/MD.0000000000049700

**Published:** 2026-07-10

**Authors:** Sih-Hsun Chiu, Tung-Ying Lee, Juo-Hau Su, Kai-Chiang Yang, Chen-Chie Wang

**Affiliations:** a Department of Medical Education, Taipei Tzu Chi Hospital, Buddhist Tzu Chi Medical Foundation, New Taipei City, Taiwan; b Department of Orthopedic Surgery, Taipei Tzu Chi Hospital, Buddhist Tzu Chi Medical Foundation, New Taipei City Taiwan; c School of Dental Technology, College of Oral Medicine, Taipei Medical University, Taipei, Taiwan; d Department of Orthopedics, School of Medicine, Tzu Chi University, Hualien, Taiwan.

**Keywords:** arthroscopic surgery, case report, fresh-frozen osteochondral allograft, navicular osteochondral lesion

## Abstract

**Rationale:**

Osteochondral lesions of the navicular bone represent diagnostically challenging pathologies in foot and ankle surgery, with limited literature on effective treatments. The concave articular surfaces are challenging to reconstruct with conventional knee-derived autografts due to substantial contour mismatch, necessitating innovative surgical approaches.

**Patient concerns:**

A 32-year-old male gardener presented with progressive left dorsal hindfoot pain following a work-related twisting injury. Initial conservative management, specifically platelet-rich plasma injection, immobilization, and activity modification, failed to provide sustained relief.

**Diagnoses:**

Magnetic resonance imaging revealed a large osteochondral defect measuring 0.9 cm × 0.7 cm × 1.1 cm on the posterior facet of the navicular bone, with a concomitant anterior talofibular ligament tear.

**Interventions:**

The patient underwent an osteochondral allograft transplantation with an anatomically matched fresh-frozen navicular allograft from the ipsilateral navicular of a cadaveric donor. The size- and contour-matching osteochondral allograft was secured into the defect using a press-fit maneuver and biodegradable screws, along with ligamentous repair.

**Outcomes:**

Postoperative imaging confirmed stable fixation and successful incorporation of the graft. At follow-up, the patient reported substantial pain relief, improved mobility, and enhanced quality of life.

**Lessons:**

This case represents one of the few documented instances of a navicular osteochondral lesion treated with anatomically matched fresh-frozen allograft transplantation. It highlights the feasibility, short-term radiologic incorporation, and clinically meaningful improvement in scores associated with this surgical approach for large navicular osteochondral lesions, emphasizing its potential value as a tailored option for younger patients with refractory lesions.

## 1. Introduction

Despite being rare, osteochondral lesions (OCLs) of the navicular bone represent a major source of chronic dorsal hindfoot pain, particularly in young and active individuals. These lesions typically arise after trauma, such as ankle sprain or direct impacts, and they involve damage to both the articular cartilage and the subchondral bone.^[1,2]^ The navicular bone’s unique anatomy and limited blood supply not only make it particularly prone to injury but also complicate treatment.^[3]^ Without proper treatment, these lesions can lead to long-term pain, reduced mobility, and instability in the talonavicular joint.^[2]^

Conservative treatment, such as immobilization, activity modification, and nonsteroidal anti-inflammatory drug administration, is typically used as the initial management approach for patients with navicular bone OCLs, particularly for smaller lesions.^[4]^ For larger lesions or refractory cases, surgical intervention becomes necessary. Surgical options for OCLs include bone marrow stimulation techniques (e.g., microfracturing) and advanced reconstruction techniques such as osteochondral autograft and allograft transplantation.^[5]^ In this report, we present the case of a navicular bone OCL treated with navicular allograft transplantation. We also review the literature on navicular OCLs.

## 2. Case presentation

This study was approved by the Institutional Review Board of Taipei Tzu Chi Hospital, Buddhist Tzu Chi Medical Foundation (Protocol No. 14-IRB119), and written informed consent was obtained from the patient for the use of clinical data and images. In September 2024, a 32-year-old male gardener presented to our orthopedic outpatient department with a chief complaint of chronic left dorsal hindfoot and intermittent lateral ankle pain (Table 1). At presentation, the patient rated his maximum level of pain as 8 out of 10 on the Visual Analog Scale. His symptoms began after a twisting injury that he sustained in March 2024, when he stepped into a pit with a depth of 50 cm at work. Initial X-ray evaluation at a local medical clinic revealed no abnormalities, and conservative management, specifically painkiller administration and 2 platelet-rich plasma injections, provided only temporary relief.

**Table 1:** Clinical timeline of a 32-year-old male patient with a navicular osteochondral lesion, from initial injury to postoperative follow-up.

Time point	Event
March 2024	Work-related twisting injury (stepped into a 50-cm pit)
March–August 2024	Conservative management: painkillers, bandage taping, 2 PRP injections
September 2024	Presentation to our department; plain radiography and MRI performed; preoperative assessment (AOFAS 14, SF-12 PCS 23.5, MCS 46.1)
December 2024	Navicular osteochondral allograft transplantation and ATFL repair
December 2024–February 2025	Non-weight-bearing for 8 wk, followed by partial weight-bearing in a walking boot for 4 wk
March 2025 (3-mo follow-up)	Plain radiographs confirmed graft incorporation; functional assessment: AOFAS 72, SF-12 PCS 48.0, MCS 48.2
June 2025 (6-mo follow-up)	Plain radiographs and MRI confirmed stable graft positioning; patient ambulating independently

AOFAS = American Orthopaedic Foot & Ankle Society, ATFL = anterior talofibular ligament, MCS = Mental Component Summary, MRI = magnetic resonance imaging, PCS = Physical Component Summary, PRP = platelet-rich plasma, SF-12 = 12-item short form health survey.

Notably, the pain experienced by the patient, which he described as both dull and sharp, was episodic and occurred every 2 to 3 days. During episodes, the intensity of pain reached 8 out of 10 on the Visual Analog Scale and required complete rest for an entire day. After treatment with painkillers and bandage taping, the intensity of pain temporarily decreased to 5 or 6 out of 10 but substantially increased when the patient walked on uneven surfaces. Despite his symptoms, the patient continued working as a gardener, walking approximately 5 km daily. Over time, the swelling and tenderness over the dorsal hindfoot progressively worsened.

**Figure 1.**
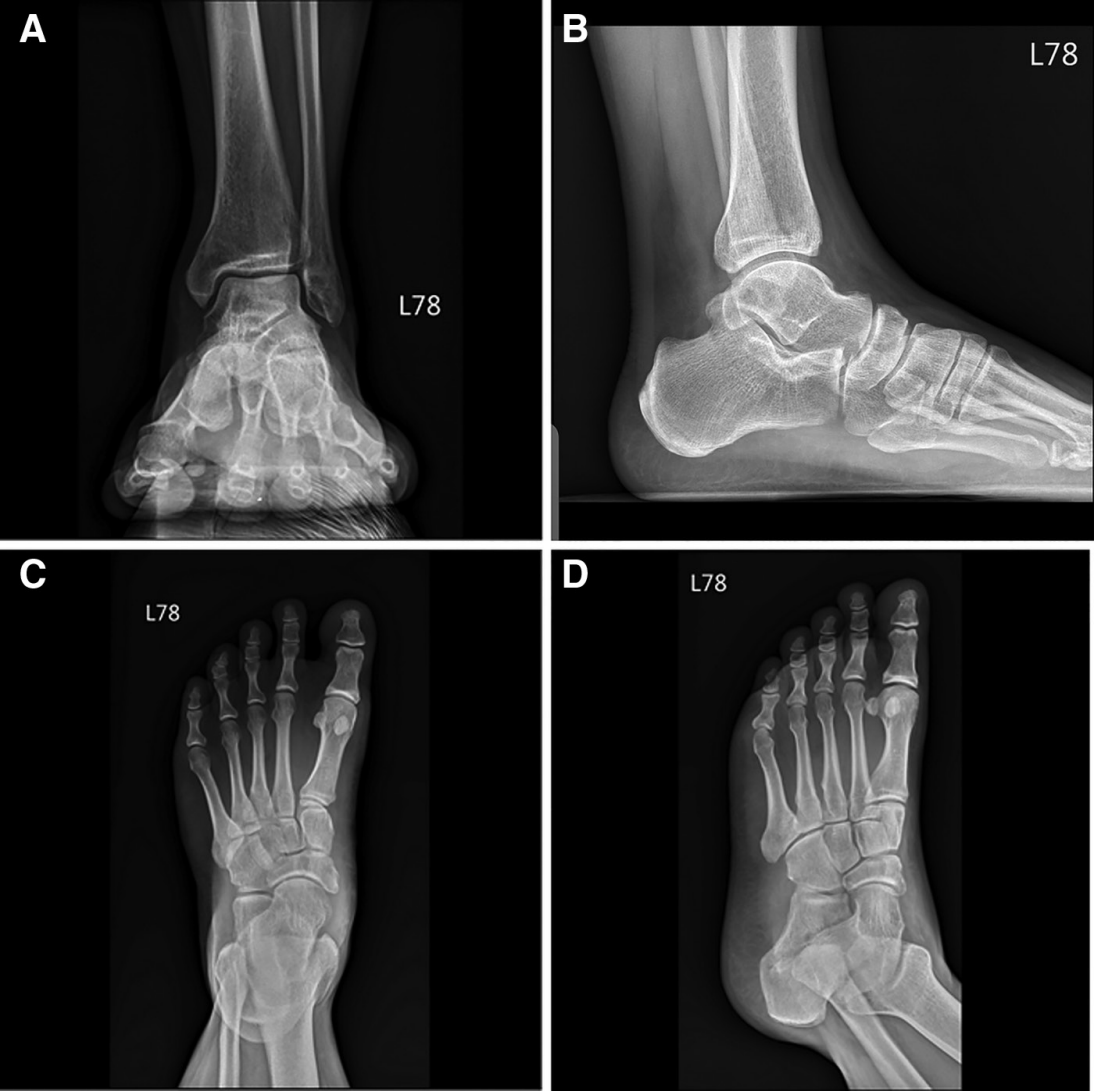
Initial plain radiographs of the left ankle and foot. Preoperative (A) anteroposterior view and (B) lateral view of the left ankle, along with (C) anteroposterior view and (D) lateral view of the left foot, demonstrating cortical irregularity of the navicular bone and a lucent lesion at its posterior facet, consistent with an osteochondral lesion.

**Figure 2.**
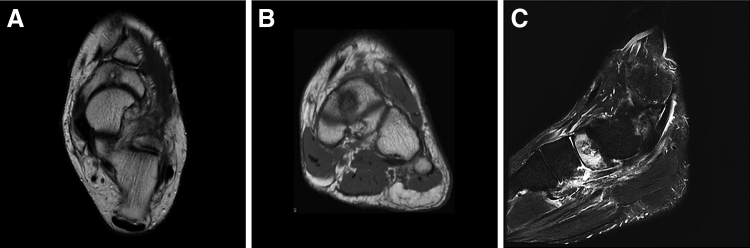
Preoperative magnetic resonance imaging of the left ankle. (A) T2 axial view, (B) T1 coronal view, and (C) sagittal T2 fast spin echo view. Images depict an osteochondral defect measuring 0.9 cm × 0.7 cm × 1.1 cm on the posterior facet of the navicular bone.

The patient had no notable medical history, including surgical interventions. Physical examination at our department revealed localized tenderness over the dorsum of the left hindfoot. Plain radiography indicated the presence of an osteochondral defect in the navicular bone (Fig. 1). The patient’s laboratory test results, including those for tumor markers and inflammatory indices, were all within normal limits. Magnetic resonance imaging of the lower extremities confirmed an osteochondral defect measuring approximately 0.9 cm × 0.7 cm × 1.1 cm on the posterior facet of the navicular bone (Fig. 2). Magnetic resonance imaging also revealed an anterior talofibular ligament (ATFL) tear, which may contribute to the patient’s chronic pain. Preoperative assessment revealed major functional limitations, with an American Orthopaedic Foot & Ankle Society (AOFAS) Ankle-Hindfoot score of 14 out of 100. It also revealed an 12-item short form health survey (SF-12) Physical Component Summary (PCS) score of 23.5 and an SF-12 Mental Component Summary (MCS) score of 46.1, indicating reduced physical function and quality of life.

Because of the failure of conservative management, surgical intervention with navicular allograft transplantation was recommended. After the procedure, risks, and potential complications were discussed with the patient, he consented to proceed and was admitted for further management.

## 3. Surgical procedure

**Figure 3.**
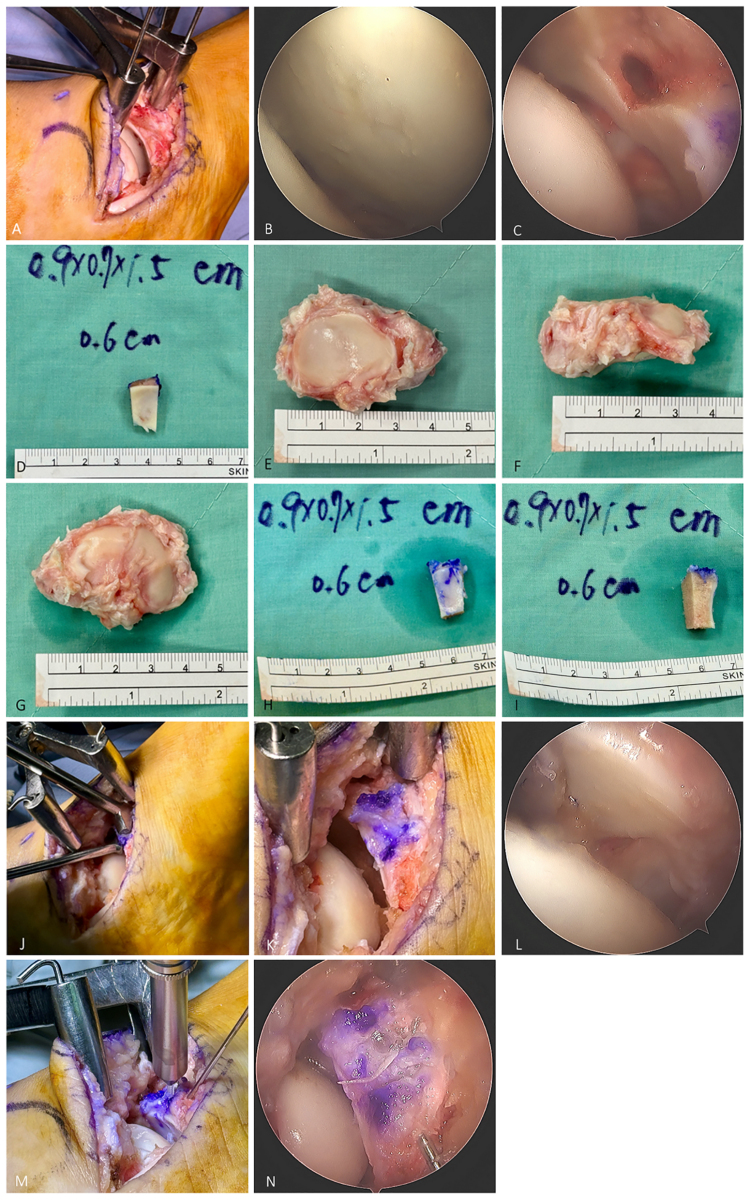
Operative images of navicular allograft transplantation procedure. (A) Dorsomedial incision over the hindfoot providing full visualization of the posterior navicular osteochondral defect. (B) Arthroscopic view of the lesion site. (C) Lesion exposed, and wound debrided to prepare the site for the allograft. (D) Bone fragment resected from the primary lesion site. (E–G) Three different facets of the fresh-frozen osteochondral allograft. (H, I) Harvested navicular allograft, measuring approximately 0.9 cm × 0.7 cm × 1.5 cm, with a smallest thickness of 0.6 cm. (J) Allograft secured into the prepared defect through a press-fit technique. (K, L) Allograft shaped to match the recipient defect and confirmed to fit through arthroscopy. (M, N) Allograft fixation using 2 biodegradable 2-mm screws (OTPS type; Inion). OTPS = orthopedic trauma plating system.

The surgical approach followed in the present case involved a carefully coordinated osteochondral allograft transplantation. In this procedure, a fresh-frozen navicular osteochondral allograft was used. The graft was procured from an accredited musculoskeletal tissue bank operating under Taiwan’s Human Organ Transplant Act, ensuring that no commercial trade in human tissues was involved. The processing procedures complied with the guidelines outlined by the Taiwan Tissue Bank Association and the American Association of Tissue Banks.^[6]^ Under general anesthesia, the patient was placed in a supine position, and dorsomedial and dorsocentral arthroscopic portals were created. A 2.3-mm, 30° scope was inserted through the dorsomedial portal for viewing. The dorsocentral portal was designated as a working portal. Careful debridement of the frayed tissue over the navicular cartilage lesion site was conducted (Fig. 3A). A dorsomedial incision over the talonavicular joint was made to provide full visualization of the navicular osteochondral defect (Fig. 3B). A Hintermann retractor was used to facilitate the exposure of the talonavicular joint. Once the joint was exposed, further debridement and resection of the whole lesion were performed, revealing a large bone defect underneath the OCL (Fig. 3C). During the procedure, the bone fragment resected from the primary lesion site was examined and found to measure approximately 0.9 cm × 0.7 cm × 1.5 cm, and a small, deeper bony defect was also found (Fig. 3D). After the lesion was removed, the created recipient bed was further drilled by a 1.25-mm Kirschner wire to facilitate the recruitment of progenitor cells from the subchondral navicular bone.

**Figure 4.**
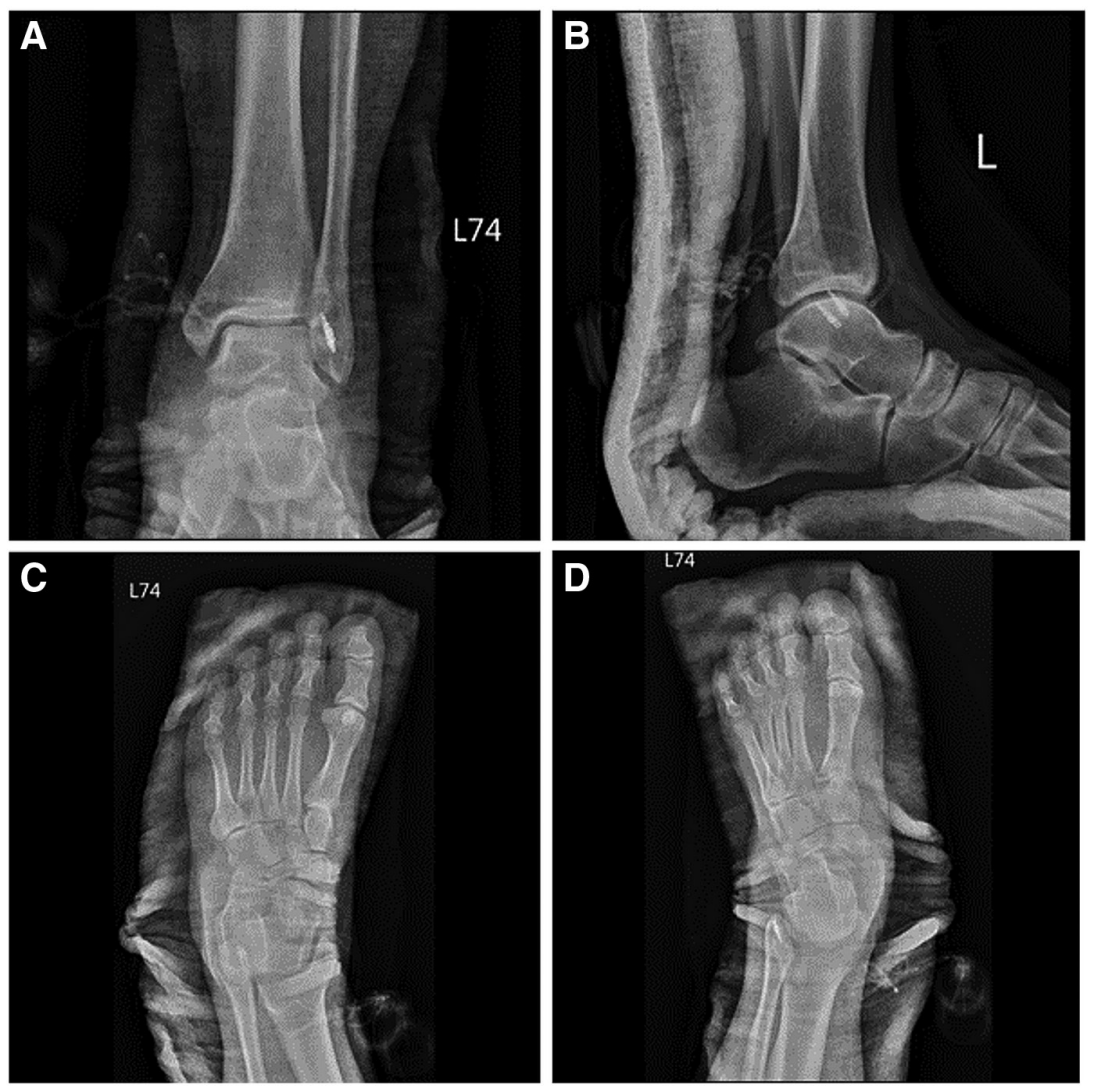
Postoperative plain radiographs of the left ankle and foot. (A) Anteroposterior view and (B) lateral view of the left ankle, and (C) anteroposterior view and (D) lateral view of the left foot, demonstrating appropriate placement and alignment of the navicular osteochondral allograft within the middle concave area. The graft demonstrates close integration with the surrounding bone structures, with no evidence of displacement or gaps. Additionally, 2 suture anchors are visible in the fibula, placed during anterior talofibular ligament repair.

The small bone defect was filled with morselized bone allografts. A fresh-frozen navicular allograft obtained from the ipsilateral navicular of a cadaveric donor was selected for this procedure (Fig. 3E–G). The same location-, size-, and contour-matching osteochondral allograft was subsequently fashioned to reconstruct the recipient defect and secured into the major osteochondral defect with a press-fit maneuver (Fig. 3H–J). Once the fit was arthroscopically confirmed, the allograft was fixed in place with 2 biodegradable 2-mm screws (orthopedic trauma plating system type; Inion, Tampere, Finland) to ensure stability and enable natural graft integration with the host tissue (Fig. 3K–3N). After grafting, intraoperative fluoroscopy confirmed proper graft placement and alignment (Fig. 4). After the wound was irrigated with sterile saline solution, the incision was closed in layers, ensuring careful approximation of soft tissue. In addition to navicular reconstruction, concomitant lateral ankle pathology was also addressed. An arthroscopic evaluation identified a complete tear of the ATFL, which was repaired using suture anchors (2.8 mm TWINFIX; Smith Nephew, Andover) combined with a Gould modification involving advancement and reinforcement of the inferior extensor retinaculum to restore ankle stability. A sterile dressing was then applied, and the patient’s foot was immobilized in a short-leg splint to provide support during the initial recovery phase. The patient was advised not to bear weight on the foot for 8 weeks, with partial weight bearing permitted for 4 weeks while wearing a walking boot. Strenuous sports activity was permitted after 6 months of physical therapy.

## 4. Results

At the 3-month postoperative follow-up in our orthopedic clinic, plain radiographs of the patient’s left ankle demonstrated radiographic evidence of graft incorporation without displacement or collapse at the primary OCL site. Clinically, the patient’s functional outcomes substantially improved. His SF-12 PCS and MCS scores increased to 48.0 and 48.2, respectively, both approaching the general population norm of 50, demonstrating a marked improvement over the preoperative assessment scores. The patient’s AOFAS Ankle-Hindfoot score also substantially increased from 14 preoperatively to 72 postoperatively.

**Figure 5.**
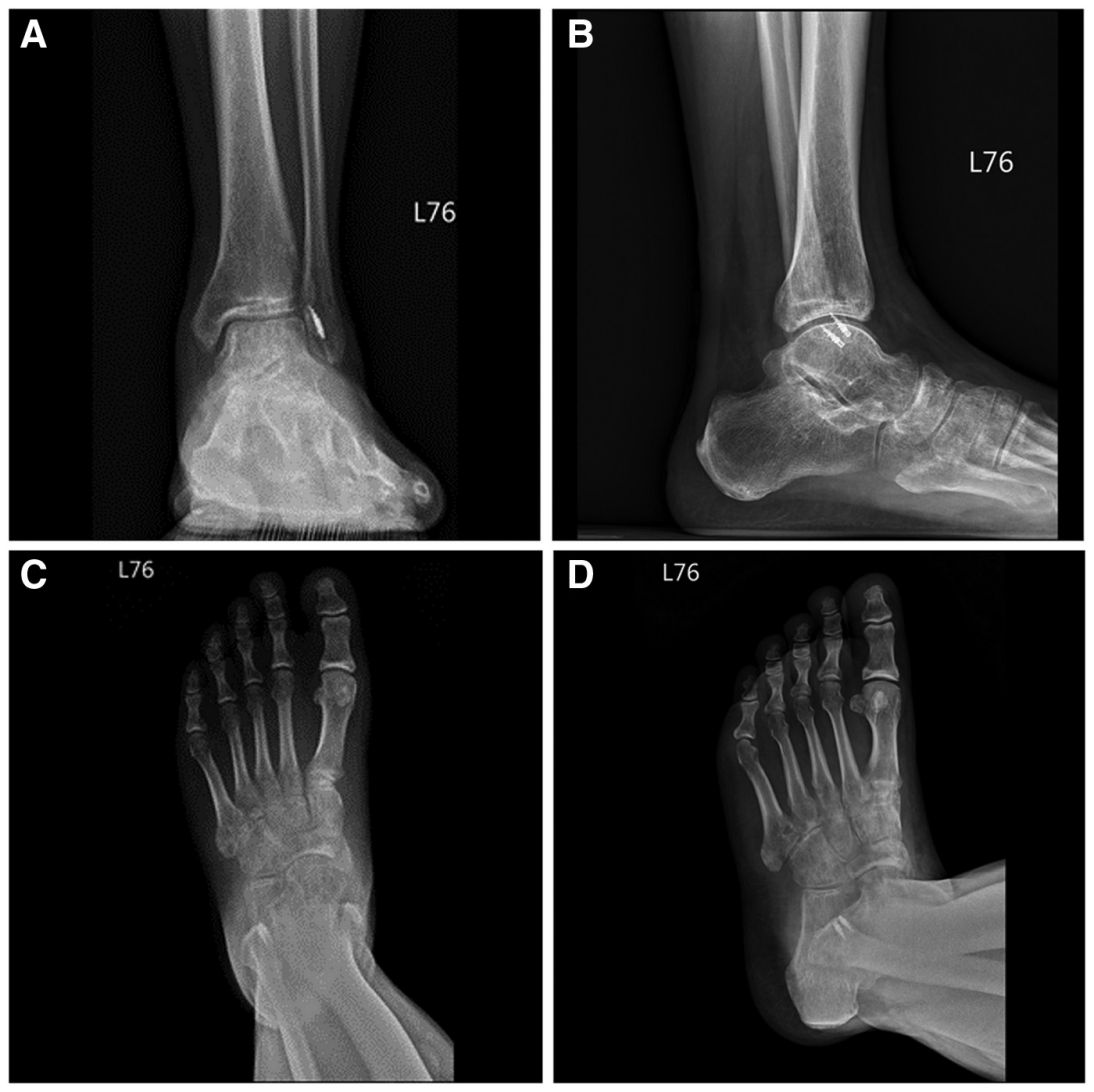
Postoperative 6-mo plain radiographs of the left ankle and foot. (A) Anteroposterior view and (B) lateral view of the left ankle, and (C) anteroposterior view and (D) lateral view of the left foot, demonstrating stable graft positioning and integration.

**Figure 6.**
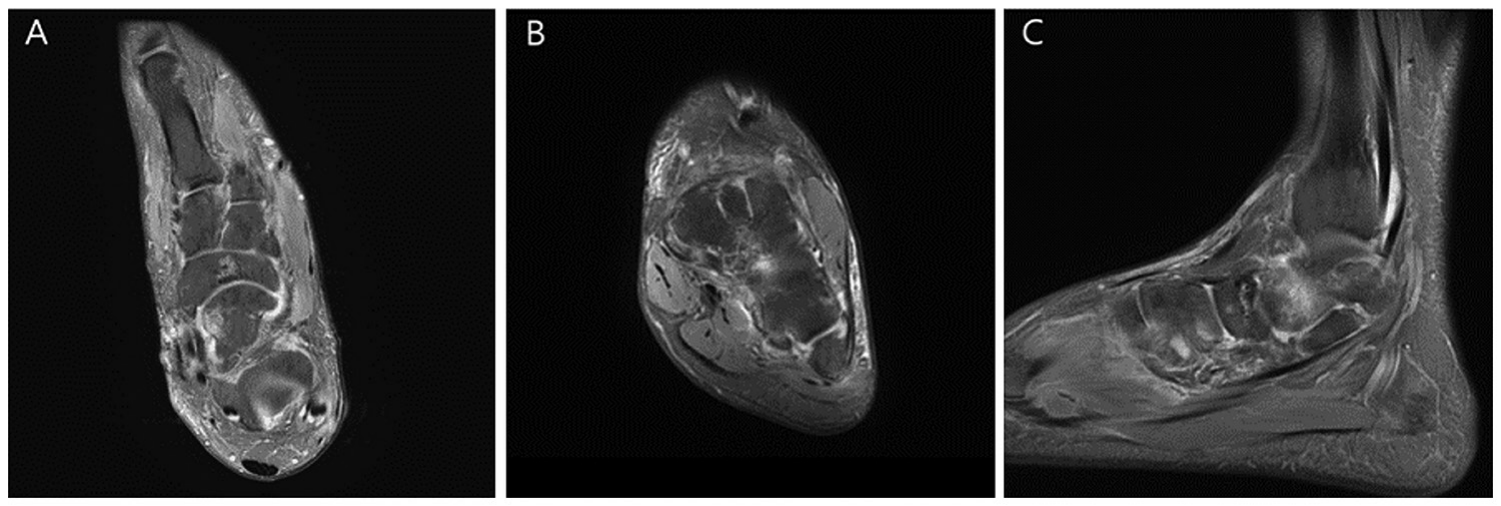
Postoperative T2 magnetic resonance imaging of the left ankle. (A) T2 axial view, (B) T1 coronal view, and (C) sagittal proton density fast spin echo view. These postoperative images demonstrate radiographic evidence of graft incorporation without evidence of displacement, collapse, or graft resorption.

At the latest 6-month follow-up, plain radiographs confirmed stable graft positioning (Fig. 5). Magnetic resonance imaging of the left ankle (Fig. 6) revealed satisfactory graft incorporation into the host bone and cartilage, with no evidence of displacement, collapse, or graft resorption. No major bone marrow edema or surrounding soft tissue abnormalities suggestive of complications were observed. The patient can ambulate independently without requiring any assistive devices.

## 5. Discussion

OCLs of the navicular bone are rare and not yet fully understood.^[7]^ Historically, these lesions have been categorized as osteochondritis dissecans. The broader term of osteochondral lesions that is currently used involves a range of injuries to cartilage and subchondral bone, including those of traumatic, ischemic, or degenerative origins.^[4]^

OCLs are often observed in athletes who engage in high-impact sports. Repetitive stress from activities such as jumping and sprinting places a high load on the navicular bone, particularly in its relatively hypovascular regions, leading to microtrauma and initiating lesion formation.^[8]^ Patients with OCLs may experience chronic talonavicular joint instability, which leads to recurrent sprains and joint dysfunction. Radiographically, OCLs often present as focal lucency, sclerosis, or cortical depression, which are essential for accurate diagnosis.^[9]^ The pathophysiology underlying these lesions involves compromised vascularity to the subchondral bone, which hinders natural healing processes and may result in persistent pain and joint degeneration.^[7]^ This lack of spontaneous recovery underscores the importance of effective therapeutic interventions, particularly in cases in which conservative management does not provide symptom relief. In these cases, surgical approaches aimed at restoring structural integrity and function become essential for optimal patient outcomes.

Surgical management of navicular OCLs has been the subject of extensive investigation. Common surgical approaches include iliac crest bone graft transplantation, flowable collagen use with an iliac crest bone marrow aspirate concentrate and fibrin glue, micronized cartilage matrix utilization, and osteochondral autograft transplantation.^[7,8,10,11]^ Kanazawa et al^[10]^ reported obtaining favorable outcomes with iliac crest bone grafts for OCLs with stress fractures. However, they indicated that the procedure required major joint resection, which may have affected the mechanical properties of the joint. Keller et al^[11]^ developed a minimally invasive approach that involves arthroscopy to introduce flowable collagen with a bone marrow aspirate concentrate and fibrin glue. They reported favorable outcomes in smaller lesions with limited subchondral damage. Togher et al^[7]^ demonstrated the efficacy of a micronized cartilage matrix with platelet-rich plasma in facilitating rapid recovery and high functional scores, particularly in patients with smaller defects who required surrounding cartilage preservation.

In our case, the patient reported a pain level of 8 on the Visual Analog Scale, an AOFAS Ankle-Hindfoot score of 14 out of 100, an SF-12 PCS score of 23.5, and an SF-12 MCS score of 46.1. After discussing the potential benefits and risks with the patient, we decided to proceed with navicular osteochondral allograft transplantation. This procedure aims to restore joint function by replacing damaged bone and cartilage with viable donor tissue, thereby promoting the long-term recovery of the navicular bone’s structural integrity and minimizing the hindfoot region’s osteoarthritis risk.

Allograft transplantation has several advantages. For example, it eliminates the need for tissue harvest from the patient, thereby addressing the complications associated with donor site morbidity.^[12]^ This approach is particularly useful for younger patients who may require multiple surgical procedures throughout their life. Furthermore, allografts can be customized to align with the precise dimensions of the target defect, ensuring an accurate fit and facilitating the treatment of larger defects, which are often challenging to address with alternative techniques.^[12]^ In our case, the OCL was located at the posterior facet of the navicular bone, which has a concave surface. The knee joint is the most common donor site for osteochondral autografts. This site’s peripheral non-weight-bearing articular surface has a convex shape not suitable for navicular OCL. Navicular allografts can provide a similar contour to articular surfaces, which can be easily achieved in cases of navicular OCLs through a press-fit maneuver without excessive grafting. Allografting is a single-stage procedure that may allow patients to resume their full activity within a year, thereby demonstrating the potential to facilitate rapid rehabilitation. Research has indicated that allografting results in high patient satisfaction and functional restoration, making it a reliable option for the management of osteochondral defects.^[13]^

While concomitant ATFL repair was performed in this case, we attribute the predominant source of chronic dorsal hindfoot pain to the navicular osteochondral lesion rather than the lateral ligamentous injury. This interpretation is supported by several clinical observations: first, the patient’s pain was localized primarily to the dorsal hindfoot rather than the lateral ankle; second, the ATFL tear was identified incidentally on MRI as an additional finding and was not associated with the hallmark symptoms of lateral ankle instability, such as recurrent sprains or giving-way episodes; and third, the degree of functional limitation reflected by the preoperative AOFAS score of 14 was disproportionate to what would be expected from isolated ligamentous insufficiency alone. Nevertheless, we acknowledge that ATFL repair likely contributed to overall stability and may have facilitated postoperative rehabilitation and functional recovery. Prospective studies incorporating patient-reported outcome measures specific to each pathology would be needed to more precisely delineate the independent contributions of each intervention.

The patient reported that prior to surgery, the episodic yet severely debilitating pain profoundly disrupted his daily work and quality of life as a gardener. He expressed initial concern regarding the novelty of the allograft procedure; however, after a thorough discussion of the risks, benefits, and alternatives with the surgical team, he felt confident in proceeding with surgery. Postoperatively, he described the recovery as gradual but encouraging, noting meaningful improvements in pain and mobility within the first 3 months. At the 6-month follow-up, he reported satisfaction with the outcome and expressed his ability to return to light occupational activities, which he considered a significant milestone in his recovery.

The 6-month follow-up period is a limitation of this case report. While our short-term results are promising, long-term graft durability, the risk of talonavicular degenerative changes, and overall long-term effectiveness require validation through larger case series and longer follow-up periods.

## 6. Conclusion

This case report highlights a frequently underdiagnosed condition in patients presenting with refractory dorsal hindfoot discomfort following unsuccessful conservative interventions. When clinicians encounter young, active patients with persistent navicular region pain and large osteochondral defects on imaging, anatomically matched fresh-frozen navicular allograft transplantation using a press-fit technique should be considered. In cases with large navicular OCLs requiring precise anatomical restoration, this approach represents a feasible surgical option with favorable functional outcomes, as evidenced by the dramatic AOFAS score improvement (14–72) and the achievement of substantial pain relief, improved mobility, and enhanced quality of life.

## Author contributions

**Conceptualization:** Chen-Chie Wang.

**Data curation:** Sih-Hsun Chiu, Tung-Ying Lee, Juo-Hau Su, Chen-Chie Wang.

**Formal analysis:** Sih-Hsun Chiu, Tung-Ying Lee, Juo-Hau Su, Chen-Chie Wang.

**Visualization:** Sih-Hsun Chiu, Tung-Ying Lee, Juo-Hau Su, Kai-Chiang Yang.

**Writing – original draft:** Sih-Hsun Chiu, Tung-Ying Lee, Juo-Hau Su, Kai-Chiang Yang, Chen-Chie Wang.

**Writing – review & editing:** Sih-Hsun Chiu, Tung-Ying Lee, Juo-Hau Su, Kai-Chiang Yang, Chen-Chie Wang.
